# Supplementary material for: Dynamic molecular network analysis of iPSC-Purkinje cells differentiation delineates roles of ISG15 in SCA1 at the earliest stage
Source: Commun Biol. 2024 Apr 9;7:413. doi: 10.1038/s42003-024-06066-z (PMC11003991; doi:10.1038/s42003-024-06066-z)
Supplement: Supplementary file 2 — Supplementary Information [file 42003_2024_6066_MOESM2_ESM.pdf]

Supplementary Information

Dynamic molecular network analysis of iPSC-Purkinje cells differentiation delineates roles of ISG15 in SCA1 at the earliest stage

Hidenori Homma[1,#], Yuki Yoshioka[1,#], Kyota Fujita[1,2,#], Shinichi Shirai[3], Yuka Hama[3], Hajime Komano[4], Yuko Saito[5], Ichiro Yabe[3], Hideyuki Okano[4], Hidenao Sasaki[3], Hikari Tanaka[1,$], and Hitoshi Okazawa[1,$]

1: Department of Neuropathology, Medical Research Institute, Tokyo Medical and Dental University, 1-5-45, Yushima, Bunkyo-ku, Tokyo 113-8510, Japan.

2: Research Center for Child Mental Development, Kanazawa University, 13-1 Takaramachi, Kanazawa-shi, Ishikawa, 920-8640, Japan.

3: Department of Neurology, Faculty of Medicine, Graduate School of Medicine, Hokkaido University, Kita 15, Nishi 7, Kita-ku, Sapporo 060-8638, Japan.

4: Department of Physiology, Keio University School of Medicine, 35 Shinanomachi, Shinjuku-ku, Tokyo 112-0012, Japan.

5: Department of Neuropathology, Tokyo Metropolitan Institute of Gerontology, 35-2 Sakae-cho, Itabashi-ku, Tokyo, 173-0015, Japan.

#: Contributed equally

$: Corresponding author

(okazawa.npat@mri.tmd.ac.jp, tanaka.npat@mri.tmd.ac.jp)

a

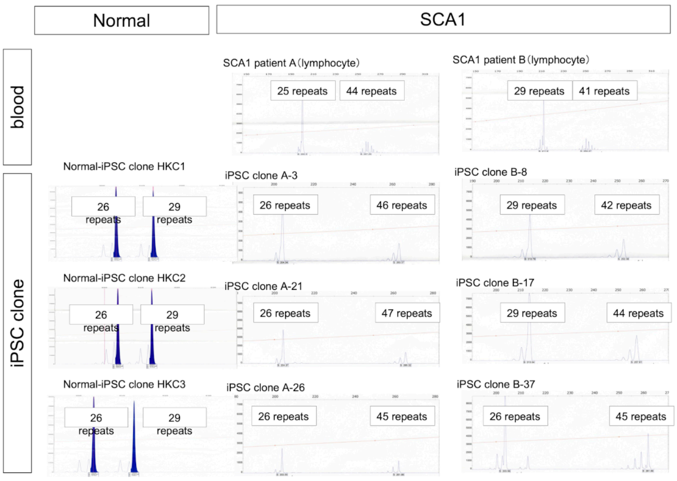

Sample			Repeat sequence	Repeat number
Healthy	201B7<br>36y<br>Female		$(CAG)_{12}$CAT CAG CAT$(CAG)_{14/15}$	–<br>(29/30)
	HKC<br>59y<br>Male	1	$(CAG)_{13}$CAT CAG CAT$(CAG)_{10/13}$	26/29
		2	$(CAG)_{13}$CAT CAG CAT$(CAG)_{10/13}$	26/29
		3	$(CAG)_{13}$CATCAGCAT$(CAG)_{10/13}$	26/29
SCA1	SCA1A<br>46y<br>Female	Peripheral blood	$(CAG)_{13}$CAT CAG CAT$(CAG)_{10}$, $(CAG)_{46}$	26/46
		3	$(CAG)_{13}$CAT CAG CAT$(CAG)_{10}$, $(CAG)_{46}$	26/46
		21	$(CAG)_{13}$CAT CAG CAT$(CAG)_{10}$, $(CAG)_{47}$	26/47
		26	$(CAG)_{13}$CAT CAG CAT$(CAG)_{10}$, $(CAG)_{46}$	26/46
	SCA1B<br>62y<br>Male	Peripheral blood	$(CAG)_{13}$CAT CAG CAT$(CAG)_{13}$, $(CAG)_{43}$	29/43
		8	$(CAG)_{13}$CAT CAG CAT$(CAG)_{13}$, $(CAG)_{44}$	29/44
		17	$(CAG)_{13}$CAT CAG CAT$(CAG)_{13}$, $(CAG)_{46}$	29/46
		37	$(CAG)_{13}$CAT CAG CAT$(CAG)_{13}$, $(CAG)_{47}$	29/47

b

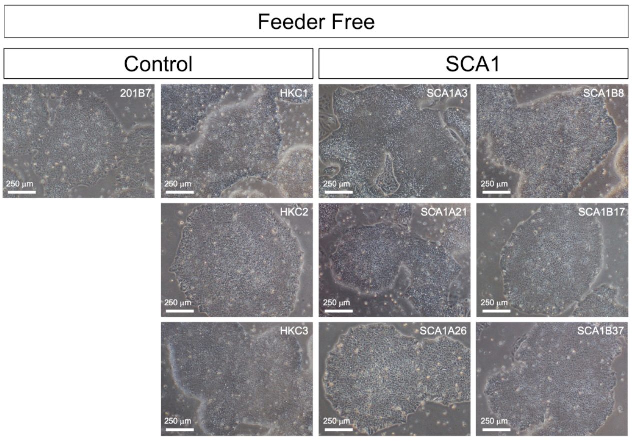

c

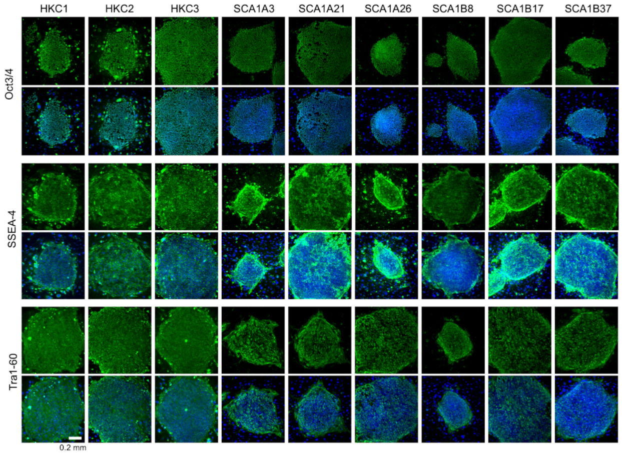

d

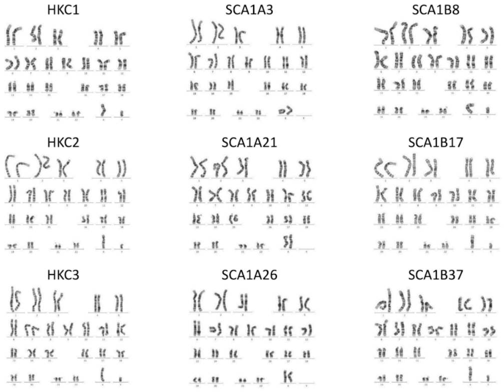

Supplementary Figure 1
Characterization of SCA1-iPSCs

a) Upper panels show fragment analysis of CAG repeat numbers in blood samples from patients or in iPSC clones established from their lymphocytes. Lower table shows CAG repeat sequences in normal and mutant allele and their repeat numbers determined by Sanger sequencing. Samples with red IDs were used for RNA-seq.
b) Morphology of iPSC clones in feeder-free culture.
c) Immunocytochemistry shows expression of pluripotent stem cell markers in normal iPSCs and SCA1 iPSC clones.
d) Chromosome analysis of iPSC clones by Gimsa staining. Abnormal chromosomes were not detected in SCA1-iPSC clones.

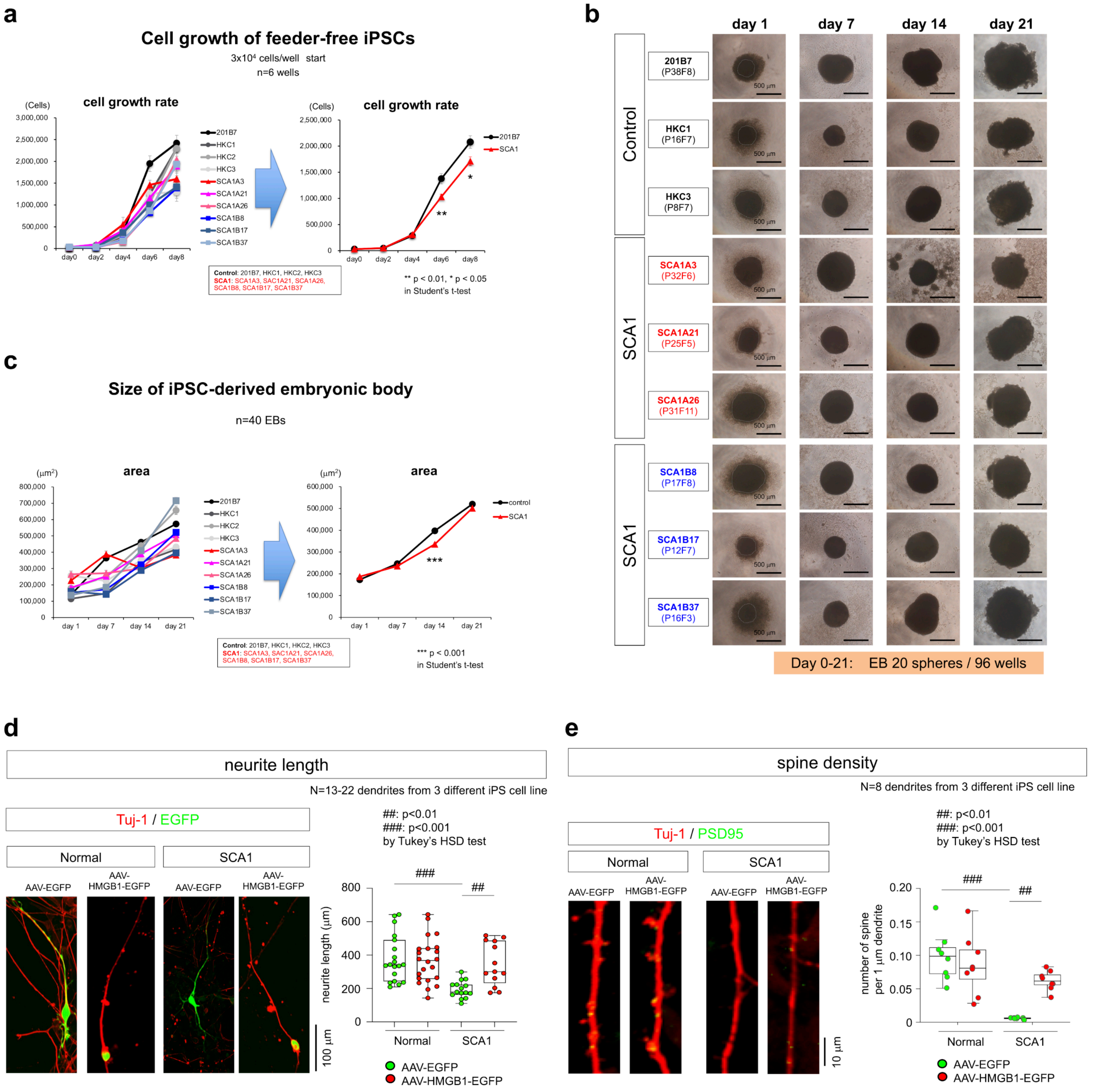

Supplementary Figure 2
Characterization of SCA1-iPSCs

a) Cell growth of iPSC clones in feeder-free culture. The two-sided Student's t-test was used for statistical comparisons in the right panel. Data are mean values ± SEM. P=0.0036 (**) at day 6 and P=0.016 (*) at day 8.
b) Growth and morphological changes of EB spheres during culture for 21 days.
c) Size increase of EB spheres. The two-sided Student's t-test was used for statistical comparisons in the right panel. Data are mean values ± SEM. $P=2.0\times10^{-11}$ (***) at day 14.
d) Neurite extension of iPSC-derived pan-neurons was evaluated after 12 days of AAV vector addition to culture medium. AAV-EGFP or AAV-HMGB1-EGFP was added at MOI of 2,000. Similar experiments were repeated three times. Tukey's HSD test was used for multiple comparisons. P=0.00027 (###) and P=0.0089 (##). The box plot shows the median and 25–75th percentile, and whiskers represent data outside the 25–75th percentile range.
e) Spine density of iPSC-derived pan-neurons was evaluated during the same period as D. Similar experiments were repeated three times. Tukey's HSD test was used for multiple comparisons. P=0.000006 (###) and P=0.0040 (##). The box plot shows the median and 25–75th percentile, and whiskers represent data outside the 25–75th percentile range.

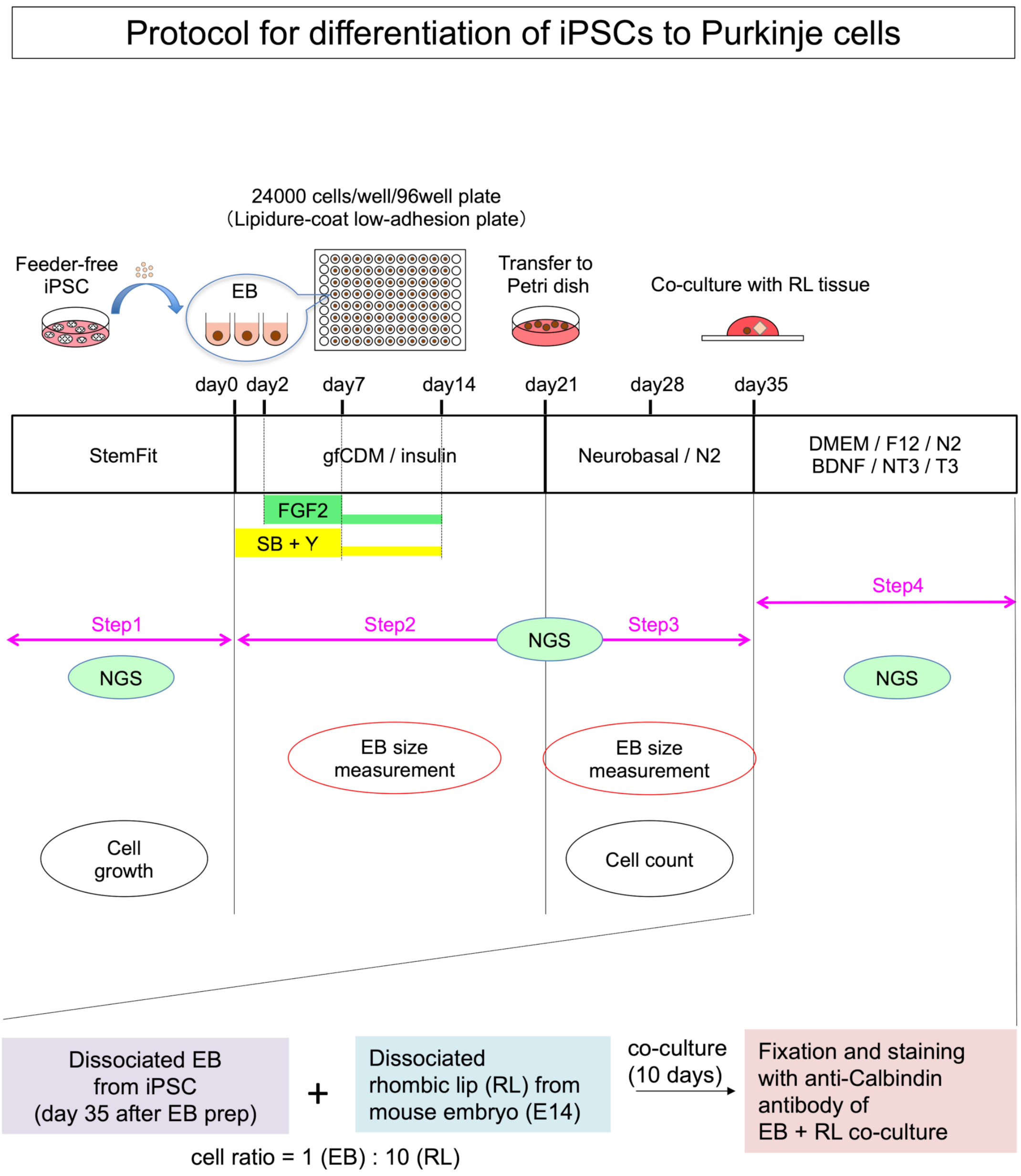

Supplementary Figure 3
Differentiation protocol of iPSCs
Protocol for differentiation of normal and SCA1 iPSCs to Purkinje cells. RL: Rhombic Lip prepared from mouse embryos at E14.

a

Observation	read length	total reads	total bases	% GC content
iPS_Normal_201B7	100	56,373,720	5,637,372,000	46.96%
iPS_Normal_HKC1	100	53,184,242	5,318,424,200	46.99%
iPS_Normal_HKC3	100	51,869,658	5,186,965,800	47.03%
iPS_SCA1A26	100	58,725,870	5,872,587,000	47.10%
iPS_SCA1B8	100	50,333,320	5,033,332,000	46.89%
iPS_SCA1B37	100	60,419,120	6,041,912,000	46.63%
EB_Normal_201B7	100	46,440,314	4,644,031,400	46.67%
EB_Normal_HKC1	100	52,031,160	5,203,116,000	46.97%
EB_Normal_HKC3	100	51,250,582	5,125,058,200	46.99%
EB_SCA1A26	100	50,260,038	5,026,003,800	46.53%
EB_SCA1B8	100	69,676,096	6,967,609,600	46.38%
EB_SCA1B37	100	49,487,394	4,948,739,400	46.00%
Purkinje_Normal_201B7	100	76,557,764	7,655,776,400	43.38%
Purkinje_Normal_HKC1	100	71,356,396	7,135,639,600	42.69%
Purkinje_Normal_HKC3	100	74,599,296	7,459,929,600	45.97%
Purkinje_SCA1A26	100	83,213,736	8,321,373,600	46.62%
Purkinje_SCA1B8	100	74,081,444	7,408,144,400	41.74%
Purkinje_SCA1B37	100	60,399,096	6,039,909,600	44.20%

b

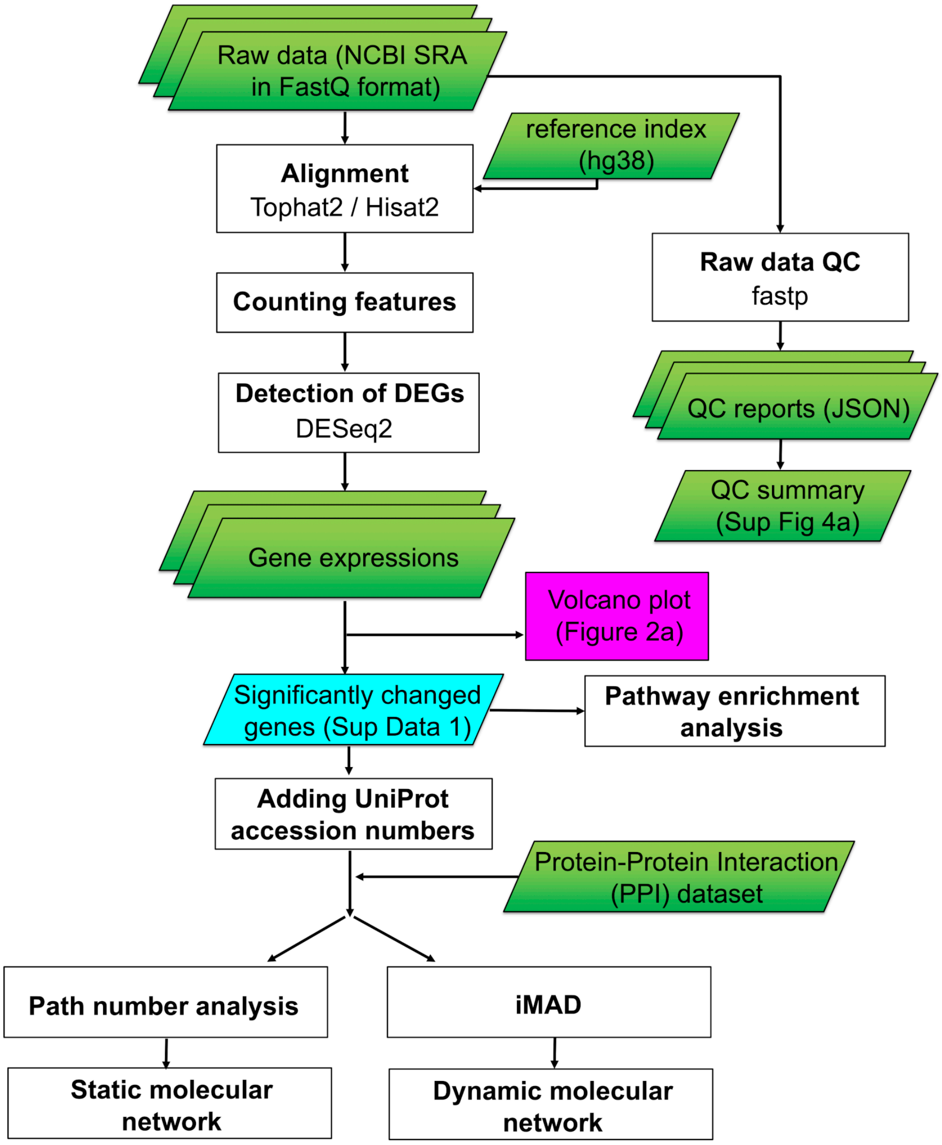

Supplementary Figure 4
Summary of RNA-seq of iPSCs
a) Summary of RNA-seq data of iPSCs at three differentiation stages. %QC is also indicated.
b) The RNA-seq data analysis pipeline is shown.

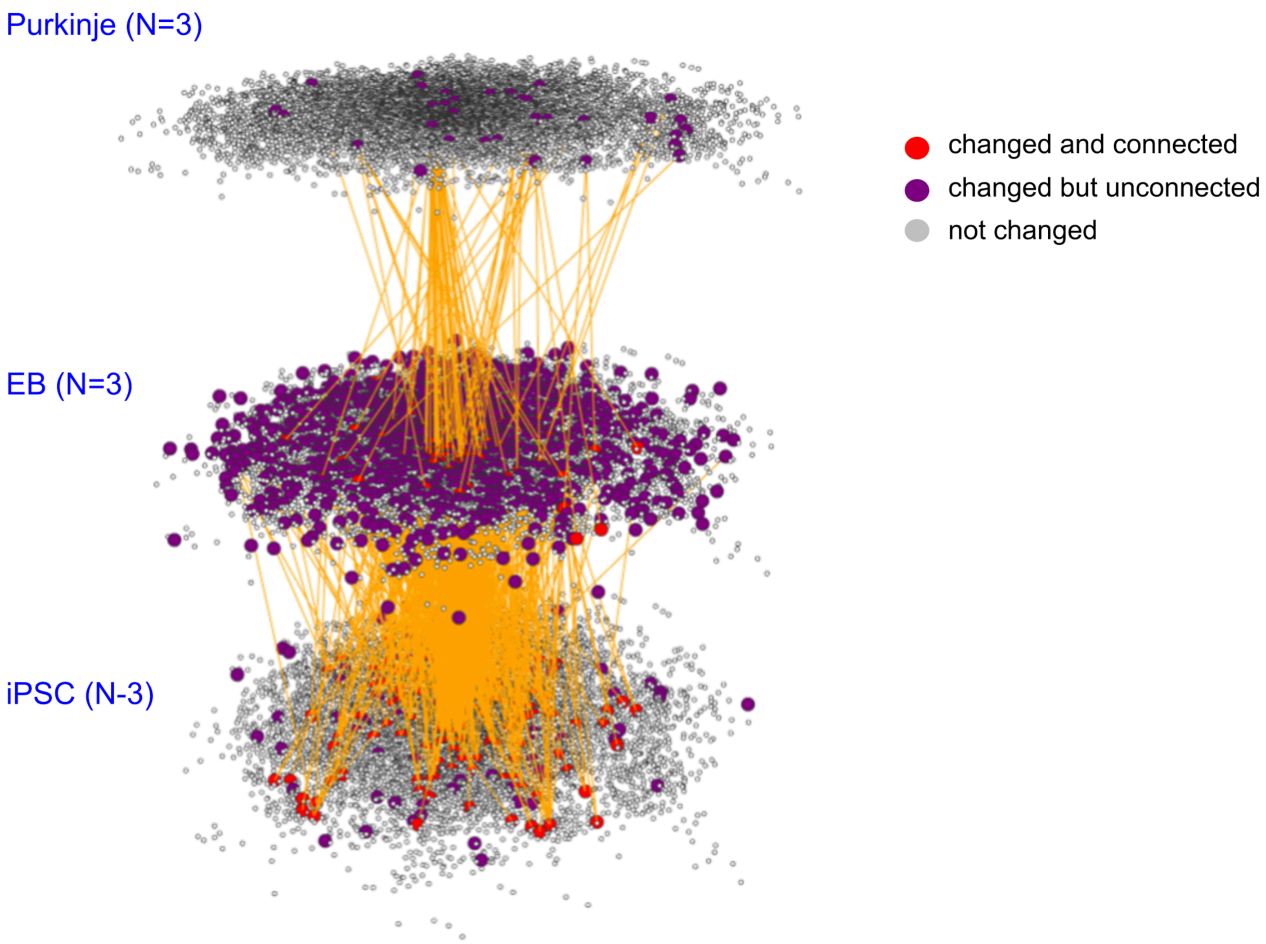

	Connected	Unconnected	Unconnected ratio
EB to Purkinje	81	2768	0.972
iPSC to EB	135	272	0.668

Supplementary Figure 5
Static molecular network of iPSCs during differentiation to Purkinje cells
Nodes with significantly changed expression at each differentiation stage were simply connected by the PPI database from the iPSC stage to the EB stage and from the EB stage to the Purkinje cell stage. Red nodes indicate proteins with PPI-based connections to the next stage, while purple nodes indicate proteins with no connections. The table shows the numbers of connected and unconnected nodes together with the ratio of connections.

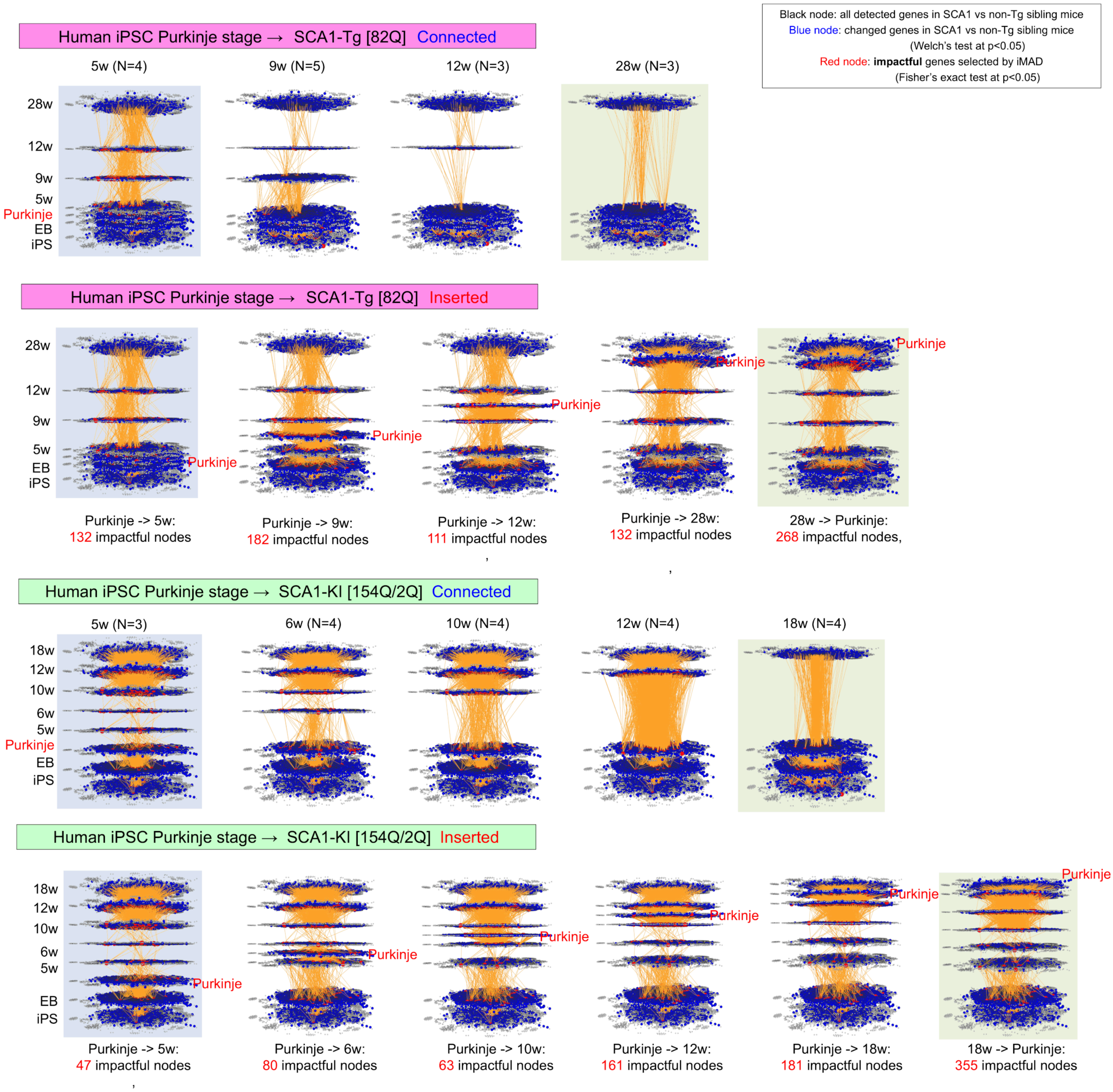

Supplementary Figure 6
Meta-analysis of dynamic molecular networks
IMAD analysis was performed with publicly available RNA-seq data of cerebellar tissues from *Atxn1*-KI and *Atxn1*-Tg mice. The mouse dynamic networks at multiple ages were connected to the Purkinje cell-stage network of iPSCs to examine their similarity. The number of mouse data is shown in Figure 5. The numbers of connected impactful nodes are shown below the network images.

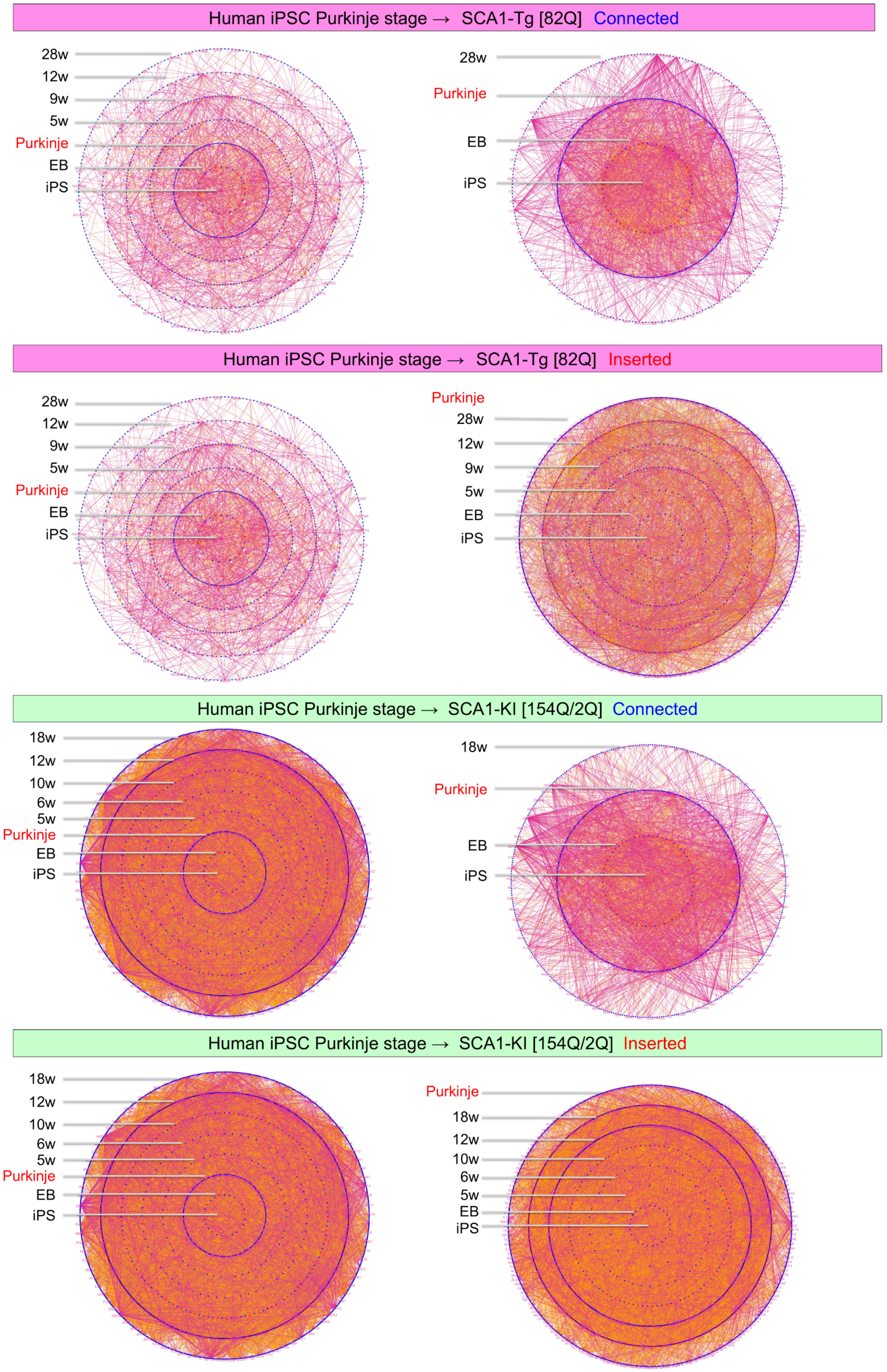

Supplementary Figure 7

Inclusion of the ISG15-relevant network in meta-analysis by iMAD

Dynamic molecular networks generated by iMAD were reorganized in concentric circle diagrams (indicated by orange lines), which included cytokine-relevant sub-networks (indicated by purple gene names and purple lines) in the *Atxn1*-KI or *Atxn1*-Tg mouse network.

Figure 1

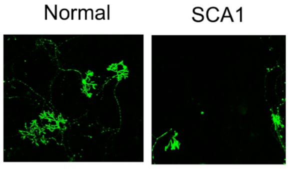

Figure 6c

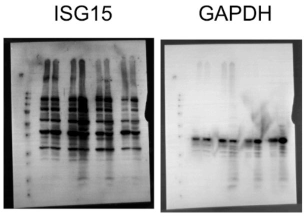

Figure 6d

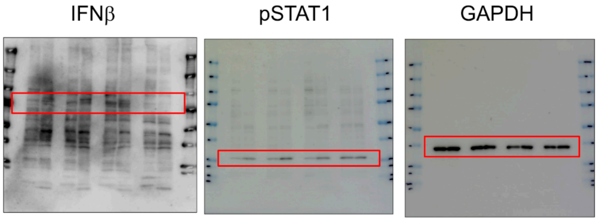

Figure 7a,b

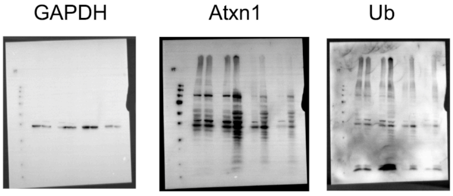

Figure 7d

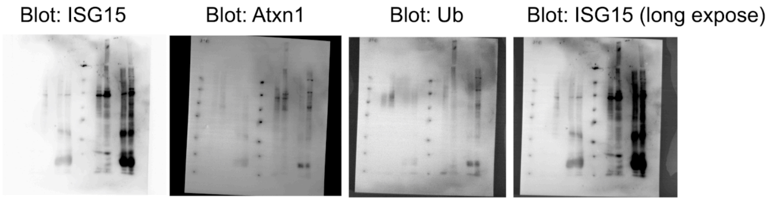

Figure 7g

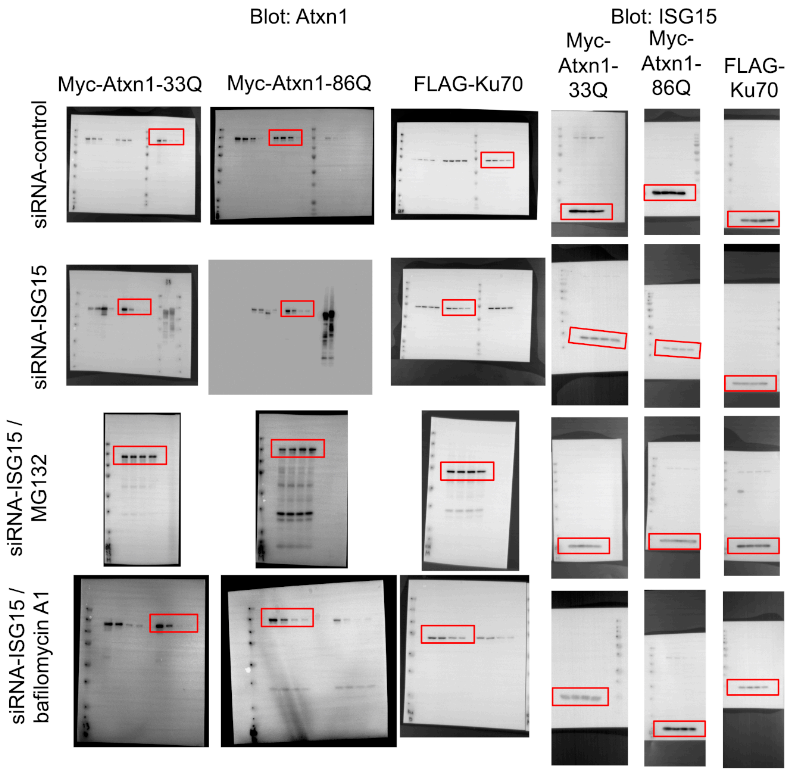

Figure 9a

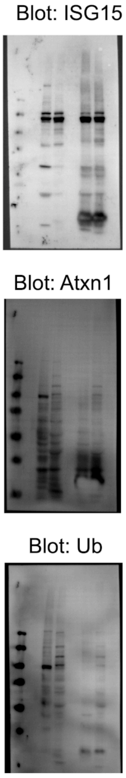

Figure 9c

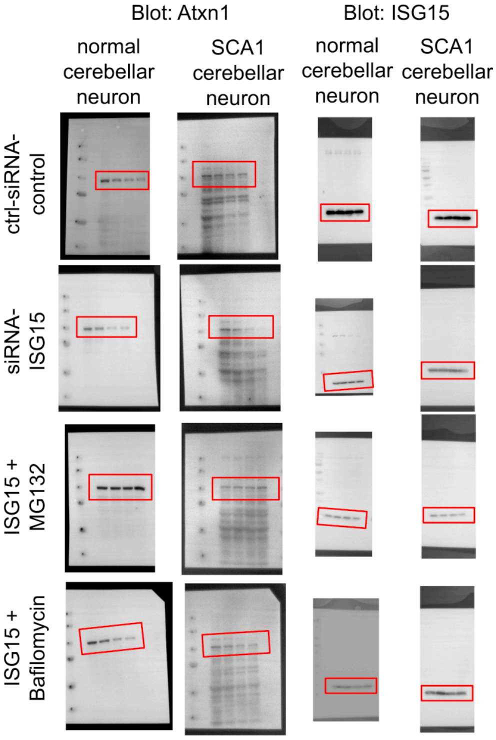

Supplementary Figure 2e

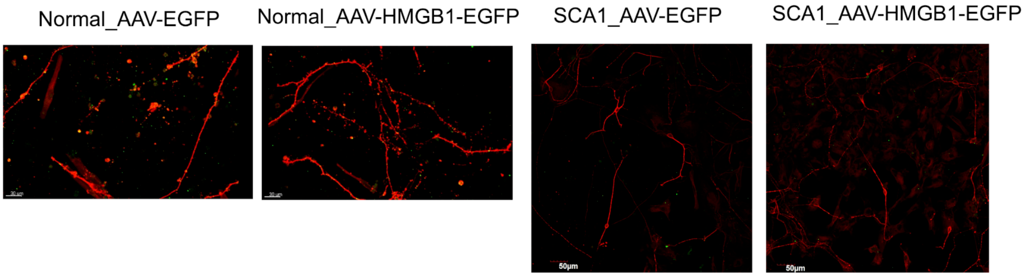

Supplementary Figure 8
Uncropped images for all figure
