# Supplementary material for: Dynamic molecular network analysis of iPSC-Purkinje cells differentiation delineates roles of ISG15 in SCA1 at the earliest stage
Source: Commun Biol. 2024 Apr 9;7:413. doi: 10.1038/s42003-024-06066-z (PMC11003991; doi:10.1038/s42003-024-06066-z)
Supplement: Supplementary file 3 — Description of Additional Supplementary Files [file 42003_2024_6066_MOESM3_ESM.pdf]

## Description of Additional Supplementary Files

**File name:** Supplementary Data 1
**Description:** List of dysregulated genes in RNA-seq analysis of SCA1-iPSCs during differentiation.

**File name:** Supplementary Data 2
**Description:** Lists of impactful genes at the iPSC, EB, and Purkinje cell stages.

**File name:** Supplementary Data 3
**Description:** Purkinje cell stage genes connected from impactful genes at iPSC stage.

**File name:** Supplementary Data 4
**Description:** Cytokine-relevant genes at PC connect from core nodes at iPSC.

**File name:** Supplementary Data 5
**Description:** Lists of nodes and edges in networks generated by iMAD-based meta-analysis.

**File name:** Supplementary Data 6
**Description:** Relationship between plasma ISG15 levels and clinical factors.

**File name:** Supplementary Data 7
**Description:** Source data for experiments.
