# Supplementary material for: Dynamic molecular network analysis of iPSC-Purkinje cells differentiation delineates roles of ISG15 in SCA1 at the earliest stage
Source: Commun Biol. 2024 Apr 9;7:413. doi: 10.1038/s42003-024-06066-z (PMC11003991; doi:10.1038/s42003-024-06066-z)
Supplement: Supplementary file 11 — Reporting Summary [file 42003_2024_6066_MOESM11_ESM.pdf]

nature portfolio

Corresponding author(s): Hitoshi Okazawa

Last updated by author(s): Oct 30, 2023

Reporting Summary

Nature Portfolio wishes to improve the reproducibility of the work that we publish. This form provides structure for consistency and transparency in reporting. For further information on Nature Portfolio policies, see our Editorial Policies and the Editorial Policy Checklist.

Statistics

For all statistical analyses, confirm that the following items are present in the figure legend, table legend, main text, or Methods section.

n/a	Confirmed	
☐	☒	The exact sample size (*n*) for each experimental group/condition, given as a discrete number and unit of measurement
☐	☒	A statement on whether measurements were taken from distinct samples or whether the same sample was measured repeatedly
☐	☒	The statistical test(s) used AND whether they are one- or two-sided<br>*Only common tests should be described solely by name; describe more complex techniques in the Methods section.*
☒	☐	A description of all covariates tested
☐	☒	A description of any assumptions or corrections, such as tests of normality and adjustment for multiple comparisons
☐	☒	A full description of the statistical parameters including central tendency (e.g. means) or other basic estimates (e.g. regression coefficient) AND variation (e.g. standard deviation) or associated estimates of uncertainty (e.g. confidence intervals)
☐	☒	For null hypothesis testing, the test statistic (e.g. F, t, r) with confidence intervals, effect sizes, degrees of freedom and P value noted<br>*Give P values as exact values whenever suitable.*
☒	☐	For Bayesian analysis, information on the choice of priors and Markov chain Monte Carlo settings
☒	☐	For hierarchical and complex designs, identification of the appropriate level for tests and full reporting of outcomes
☒	☐	Estimates of effect sizes (e.g. Cohen's *d*, Pearson's *r*), indicating how they were calculated

Our web collection on statistics for biologists contains articles on many of the points above.

Software and code

Policy information about availability of computer code

Data collection	Images were acquired with confocal microscopy (FV1200IXGP44, Olympus, Tokyo, Japan) and super-resolution microscopy (LSM980 with Airyscan 2, Zeiss, Oberkochen, Germany). Signal intensities were measured using ImageJ software (ver.1.53, NIH).
Data analysis	GraphPad Prism 8 software (GraphPad Software, La Jolla, CA, USA) was used for statistical analyses and to plot data.<br>Microsoft Excel for Microsoft 365 was used to draw graphs.

For manuscripts utilizing custom algorithms or software that are central to the research but not yet described in published literature, software must be made available to editors and reviewers. We strongly encourage code deposition in a community repository (e.g. GitHub). See the Nature Portfolio guidelines for submitting code & software for further information.

Data

Policy information about availability of data

All manuscripts must include a data availability statement. This statement should provide the following information, where applicable:

- Accession codes, unique identifiers, or web links for publicly available datasets
- A description of any restrictions on data availability
- For clinical datasets or third party data, please ensure that the statement adheres to our policy

We wrote the following sentences in "Data Availability" section.

Research involving human participants, their data, or biological material

Policy information about studies with human participants or human data. See also policy information about sex, gender (identity/presentation), and sexual orientation and race, ethnicity and racism.

Reporting on sex and gender	The sex and gender were not considered in the study design.
Reporting on race, ethnicity, or other socially relevant groupings	iPS cells were generated from Japanese patients.
Population characteristics	Human plasma samples were acquired from SCA1 patients with PCR-based genetic diagnosis or control patients without neurological disorders.
Recruitment	Described as above.
Ethics oversight	All experiments were approved by the Committees on Gene Recombination Experiments, Human Ethics, and Animal Experiments of the Tokyo Medical and Dental University (G2018-082C3, O2020-002-03, 2014-5-3 and A2021-211A).

Note that full information on the approval of the study protocol must also be provided in the manuscript.

Field-specific reporting

Please select the one below that is the best fit for your research. If you are not sure, read the appropriate sections before making your selection.

☒ Life sciences ☐ Behavioural & social sciences ☐ Ecological, evolutionary & environmental sciences

For a reference copy of the document with all sections, see nature.com/documents/nr-reporting-summary-flat.pdf

Life sciences study design

All studies must disclose on these points even when the disclosure is negative.

Sample size	No sample size calculation was performed, and the sample size were similar to those reported in previous publications, PubMed ID 29397273, 27641503 and 34980925.
Data exclusions	There are no exclusion criteria for all analysis.
Replication	Experiments were independently repeated, the numbers of biological replicates are presented in the Figures.
Randomization	Simple randomization was performed to allocate samples and/or images to researchers before analysis.<br>The selection of images from immunohistochemistry/immunocytochemisty and the actual experiments of IHC/ICC were done by different researchers. In vitro live-cell imaging were done by different researchers.<br>Western blots are repeated until the necessary N was acquired.
Blinding	The information about group allocation or samples were opened to the data analyst or image acquisition researchers after finalizing results (m ake graphs etc).

Reporting for specific materials, systems and methods

We require information from authors about some types of materials, experimental systems and methods used in many studies. Here, indicate whether each material, system or method listed is relevant to your study. If you are not sure if a list item applies to your research, read the appropriate section before selecting a response.

Materials & experimental systems

n/a	Involved in the study	
☐	☒	Antibodies
☐	☒	Eukaryotic cell lines
☒	☐	Palaeontology and archaeology
☐	☒	Animals and other organisms
☒	☐	Clinical data
☒	☐	Dual use research of concern
☒	☐	Plants

Methods

n/a	Involved in the study	
☒	☐	ChIP-seq
☒	☐	Flow cytometry
☒	☐	MRI-based neuroimaging

Antibodies

Antibodies used	Antibodies used for analyses of differentiated iPSCs were as follows: mouse anti- III-tubulin 1 (1:2000 for 16 h at 4°C, #T8660 Sigma-Aldrich, St5 (1:1000 for 16 h at 4°C, 3409, Cell Signaling Technology, Danvers, MA, USA), donkey antimouse IgG Alexa 488-conjugated (1:600 for 1 h at room temperature, #715-545-150, Jackson ImmunoResearch Laboratories, West Grove, PA, USA), and donkey antirabbit IgG Alexa488-conjugated (1:1000 for 1 h at room temperature, A-21206, Thermo Fisher Scientific), mouse anti-Calbindin (1:2000 for 16 h at 4°C, C9848, Sigma-Aldrich, St. Louis, MO, USA) and donkey antimouse IgG Alexa 488-conjugated (1:1000 for 1 h at room temperature, A-21202, Thermo Fisher Scientific).<br><br>Antibodies used for innmunohistochemistry were as follows: rabbit anti-ISG15 (1:100 for 16 h at 4°C, HPA004627, Sigma-Aldrich, St. Louis, MO, USA), mouse anti-Calbindin (1:2000 for 16 h at 4°C, C9848, Sigma-Aldrich, St. Louis, MO, USA), mouse anti-Atxn1 (1:100 for 16 h at 4°C, MABN37, Millipore, Burlington, MA, USA), mouse anti-ubiquitin (1:100 for 16 h at 4°C, #3936, Cell Signaling technology, Danvers, MA, USA), donkey anti-mouse IgG Alexa 488-conjugated (1:600 for 1 h, #715-545-150, Jackson ImmunoResearch Laboratories, West Grove, PA, USA), and donkey anti-rabbit IgG Cy3-conjugated (1:600 for 1 h, #711-165-152, Jackson ImmunoResearch Laboratories, West Grove, PA, USA).<br><br>Antibodies used for western blot analysis were as follows: rabbit anti-ISG15 (1:1000 for 3 h at room temperature, HPA004627, Sigma-Aldrich, St. Louis, MO, USA), mouse anti-GAPDH (1:3000 for 16 h at 4°C, MAB374, Merck, Darmstadt, Germany), mouse anti-Atxn1 (1:1000 for 3 h at room temperature, MABN37, Millipore, Burlington, MA, USA), mouse anti-ubiquitin (1:1000 for 16 h at 4°C, #3936, Cell Signaling Technology, Danvers, MA, USA), mouse anti-Myc (1:3000 for 1 h at room temperature, M047-3, MBL, Aichi, Japan), rabbit anti-FLAG (1:3000 for 1 h at room temperature, F7425, Sigma, St. Louis, MO, USA), sheep anti-mouse IgG HRP conjugated (1:3000 for 1 h, NA931, Cytiva, Tokyo, Japan), and rabbit anti-IgG HRP conjugated (1:3000 for 1 h, NA934, Cytiva, Tokyo, Japan).<br><br>Antibodies used for immunoprecipi9tation were as follows: rabbit anti-ISG15 antibody (aHPA004627, Sigma-Aldrich, St. Louis, MO, USA).
Validation	Information of validation of commercially available antibodies are provided on the manufacturer's websites.

Eukaryotic cell lines

Policy information about cell lines and Sex and Gender in Research

Cell line source(s)	HeLa cells were kindly gifted from Dr. Naoyuki Kataoka (Tokyo University) that were purchased from RIKEN BRC Cell Bank (Tsukuba, Japan).
Authentication	None of cell lines used were authenticated.
Mycoplasma contamination	All cell lines were negative for mycoplasma contamination.
Commonly misidentified lines (See ICLAC register)	We did not use any misidentified cell lines.

Animals and other research organisms

Policy information about studies involving animals; ARRIVE guidelines recommended for reporting animal research, and Sex and Gender in Research

Laboratory animals	Mutant Ataxin-1 knock-in mice (Sca1154Q/2Q mice) were a generous gift from Prof. Huda Y. Zoghbi (Baylor College of Medicine, Houston, TX, USA)101. C57BL/6J (BL/6) mice were used for breeding. Non-transgenic sibling mice were used as controls.<br>Mice were kept in 12 light/12 dark cycle, maintained at 20-22 degree with 40-60% humidity. Mice can access to food and water ad libitum.
Wild animals	The study did not involve any wild animals.
Reporting on sex	Both male and female mice were studied and compared in each of the experiments reported in this manuscript.
Field-collected samples	The study did not involve any samples collected from the field.
Ethics oversight	This study was performed in strict accordance with the recommendations of the Guide for the Care and Use of Laboratory Animals of the Japanese Government and National Institutes of Health. All experiments were approved by the Committees on Gene Recombination Experiments, Human Ethics, and Animal Experiments of the Tokyo Medical and Dental University (G2018-082C3, O2020-002-03, 2014-5-3 and A2021-211A).

Note that full information on the approval of the study protocol must also be provided in the manuscript.

Plants

Seed stocks	Not applicable.
Novel plant genotypes	Not applicable.
Authentication	Not applicable.
